# Design and Validation of an Experimental Setup for Evaluation of Gas Permeation in Ceramic Membranes

**DOI:** 10.3390/membranes13020246

**Published:** 2023-02-18

**Authors:** Sabrina G. M. Carvalho, Eliana N. S. Muccillo, Reginaldo Muccillo

**Affiliations:** 1 Center of Science and Technology of Materials, Energy and Nuclear Research Institute, Cidade Universitária, Av. Prof. Lineu Prestes, 2242, São Paulo 05508-000, SP, Brazil; 2 Institute of Physics, University of São Paulo, São Paulo 05508-090, SP, Brazil

**Keywords:** ceramic membranes, gas permeation setup, carbon dioxide capture

## Abstract

An experimental setup for the evaluation of permeation of gaseous species with the possibility of simultaneously collecting electrochemical impedance spectroscopy data in disk-shaped ceramic membranes was designed and assembled. It consists of an alumina sample holder with thermocouple tips and platinum electrodes located close to both sides of the sample. Water-cooled inlet and outlet gas connections allowed for the insertion of the sample chamber into a programmable split tubular furnace. Gas permeation through a ceramic membrane can be monitored with mass flow controllers, a mass spectrometer, and an electrochemical impedance analyzer. For testing and data validation, ceramic composite membranes were prepared with the infiltration of molten eutectic compositions of alkali salts (lithium, sodium, and potassium carbonates) into porous gadolinia-doped ceria. Values of the alkali salt melting points and the permeation rates of carbon dioxide, in agreement with reported data, were successfully collected.

## 1. Introduction

### 1.1. Membranes

Ceramic membranes are important components of devices for the separation/production of gaseous species, such as oxygen, hydrogen, and carbon dioxide. There is a continuous demand in novel and efficient devices for oxygen production for the application in several industrial sectors, for hydrogen to be used in clean energy production, and for the capture and separation of carbon dioxide to achieve a pollution-free environment. The electroceramics that play an important role for the success of producing those gases are oxygen ion-, proton-, and carbon dioxide-ion conductors.

#### 1.1.1. Membranes for Oxygen

Oxygen is an important commodity due to its frequent use in several industrial sectors, including in food, agriculture, medical, pharmaceutical, and metallurgical industries. Many efforts have been carried out on applied research looking for promising techniques for its production. Ceramic dual-phase composite membranes with suitable performance and stability may be used for the separation of oxygen from air, with a good selectivity for producing a high purity (>99.99%). A thorough review of all developed ceramic membranes, from the basic properties to the industrial application, may be found in reference [1].

#### 1.1.2. Membranes for Hydrogen

Hydrogen has attracted attention, mainly for its use in renewable and clean energy sources. One of the sources of its production is a mixed-conducting ceramic membrane. Water is introduced to the ceramic surface and, after dissociation, oxygen is removed, resulting in a flow of hydrogen-enriched carrier gas [2]. Low-coast proton and electron conducting dual-phase composite membranes, besides presenting high selectivity to hydrogen, show high stability [3].

#### 1.1.3. Membranes for Carbon Dioxide

One of the main sources of greenhouse gas emissions, particularly carbon dioxide, is the combustion of fossil fuels. One way to minimize this effect, while the non-pollutant generation of electricity and conversion of energy are not cost-effectively applicable, is carbon capture by the separation of carbon dioxide emitted from power plants fired with coal or natural gas, from industrial plants, and from non-electrical cars and trucks [4,5].

Ceramic membranes have been widely proposed for carbon dioxide separation processes [6,7,8,9,10,11,12]. They are two-phase composite membranes: a matrix consisting of porous oxygen ion conductor (e.g., gadolinia-doped ceria, samaria-doped ceria, yttria-stabilized zirconia) impregnated with a eutectic composition of alkali salts (Li-Na, Li-K, Na-K, and Li-Na-K carbonates), with 501, 498, 710, and 397 °C melting points, respectively [13]. Those membranes operate at the melting point of the eutectic composition for long times with reasonable carbon dioxide flow rates, ranging from 0.1 to 1.3 mL cm^−2^ min^−1^, depending on the physical characteristics of the membrane, the measured temperature, and the atmosphere of the experiments [14,15,16,17,18,19].

### 1.2. Carbon Dioxide Permeation

Permeation is an important parameter for evaluating the performance of a ceramic membrane. One of the requirements for designing ceramic membranes for carbon dioxide separation is a relatively high permeation of CO_3_^2−^ ions. The permeation mechanism is based on the surface reaction between the CO_2_ from the feed side and oxide anions, O^2−^, from the solid oxide (SO) ceramic phase to form CO_3_^2−^. The carbonate ion is transported through the molten carbonate (MC) phase to the other side of the membrane, according to the reaction below:CO_2 (gas)_ + O^2−^ _(SO)_

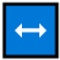
 CO_3_^2−^ _(MC)_

On the permeated side of the membrane, with low CO_2_ partial pressure, the reverse reaction occurs, releasing CO_2_ back into the gas phase. This reaction also releases the oxide anion in the reverse path through the ceramic phase, to the feed side of the membrane with a high CO_2_ partial pressure, to restart the reaction [20,21]. For the carbon dioxide to be transported through the membrane, the flow of carbonate ions needs to be balanced by a counter flow of O^2−^ ions. If one of the ionic phase resistances is higher than the other, the membrane efficiency will be limited by the most resistive phase.

The permeation flux (J) is defined as the volume of gas flowing through the membrane per unit area and unit time (mL cm^−2^ min^−1^). Permeability (mol m^−1^ s^−1^ Pa^−1^) is the permeation flux normalized by the partial pressure difference of the permeated gas and membrane thickness, while permeance (mol m^−2^ s^−1^ Pa^−1^) is normalized only for the partial pressure driving force [14,20]. Both parameters can be used to compare membrane performances at a given temperature, as they are an intrinsic characteristic of the membrane.

The CO_2_ flux depends strongly on the CO_3_^2−^ ionic conductivity through the molten carbonate mixture and the O^2−^ ionic conductivity through the ceramic phase. A commonly used molten carbonate mixture (400 °C melting point) is the ternary eutectic carbonate (Li-Na-K)_2_CO_3_ with a 43.5:31.5:25 molar ratio concentration [14,18,20,22,23,24,25,26].

The most common experimental setup for measuring CO_2_ permeation flux consists of a membrane fixed to an alumina tube, through which the feed gas is inserted and delivered to the sample [10,12,15,16,18,20,26]. This tube-sample system is placed inside another alumina or quartz tube with a thermocouple fixed to monitor the temperature and the sweep gas carrying the permeated CO_2_ to the gas analyzer, usually a mass spectrometer or a gas chromatograph.

Here, we show details of an experimental setup designed for the qualitative and quantitative measurement of the content of gaseous species that flow through single ceramic membranes, together with the possibility of simultaneously collecting data on the electrochemical impedance spectroscopy of the membrane. The analysis of the electrical properties during permeation provides information on the ionic conductivity and transport of charged species of the membrane under study. The performance of the experimental setup was ascertained by monitoring carbon dioxide permeation through gadolinium-doped ceria/lithium-sodium carbonates (GDC-LNC) dual phase composite membranes.

## 2. Materials and Methods

### 2.1. Membrane Preparation

For testing the performance of the experimental setup, two ceramic compositions were prepared: (a) alumina (α-Al_2_O_3_, Alcoa, Pittsburgh, PA, USA, 0.6 μm average particle size, 6.5 m^2^ g^−1^ specific surface area), cold pressed uniaxially at 10 MPa and isostatically at 100 MPa, followed by sintering at 1600 °C/3 h, and this dense alumina sample was used to evaluate and validate the sealing process; and (b) CeO_2_: 20 mol.% Gd_2_O_3_-Li_2_CO_3_/Na_2_CO_3_ (gadolinia-doped ceria, GDC-eutectic lithium sodium carbonate, LNC) membrane by the infiltration of molten LNC into a porous GDC ceramic pellet. The porous 20GDC (Ceramic Powder Technology AS, Tiller, Norway) matrix was prepared by tape casting using 10 wt.% of rice starch (Remy Ind., Leuven, Belgium) as a pore former and sintered at 1450 °C/5 h with low heating rates to remove all organic additives. A eutectic mixture of sodium and lithium carbonates (99.99%, Alfa Aesar, Tewksbury, MA, USA) was prepared by mixing the powders 52 mol.% Li_2_CO_3_ with 48 mol.% Na_2_CO_3_ and infiltrating it to the porous membrane at 600 °C/1 h. That membrane was attached to the alumina tube of the experimental setup to evaluate the CO_2_ permeation at different temperatures. The leakage test, using the alumina sample, was performed at 550, 610, and 665 °C, and the permeation measurement was performed at 390, 600, and 705 °C, both experiments were carried out by injecting 50% Ar-50% CO_2_ as feed gas and N_2_ as the sweep gas.

### 2.2. Experimental Setup

**Figure 1:**
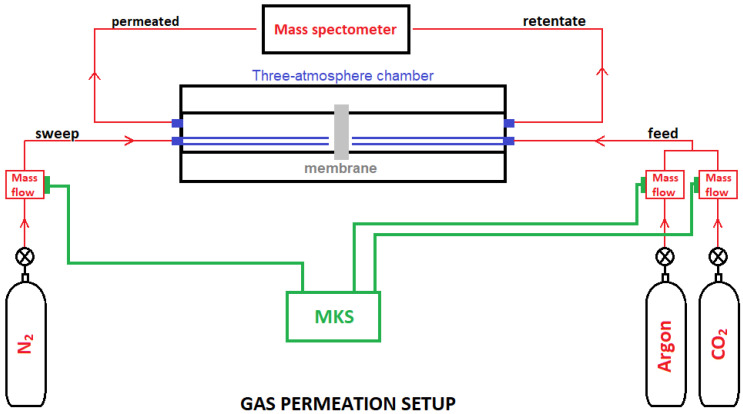
Overall structure of the components of the whole experimental setup for analysis of CO_2_ permeation through a ceramic membrane.

Figure 1 shows a drawing of the components of the experimental setup used for the analysis of CO_2_ pressure gradient-assisted permeation through a ceramic membrane. The setup consists basically of a three-atmosphere sample chamber (one gas at each side of the ceramic membrane, another gas for sweeping out any leakage at the membrane sealing cement), mass flow controllers, and a mass spectrometer. A ceramic paste containing aluminum oxide (58 wt.%), titanium dioxide (7 wt.%), and calcium oxide (35 wt.%) was mixed with water and used to attach the surface of a disk-shape membrane (see Figure 1) to the end of two alumina tubular pieces positioned in the center of a programmable split tubular furnace. After curing the ceramic sealant at 120 °C for 3 h, a selected mixture of carbon dioxide and argon (feed gas) is injected to the surface of the membrane through a 1/2” alumina tube. A similar 1/2” alumina tube is used for introducing nitrogen (sweep gas) to the other surface of the membrane. The gas permeated to the other side of the membrane is quantitatively and qualitatively analyzed with a mass spectrometer (Thermostar^®^, Pfeiffer Vacuum, Germany). Controlled fluxes of gases are injected with mass flow controllers (MKS Instruments, Inc., Andover, MA, USA).

**Figure 2:**
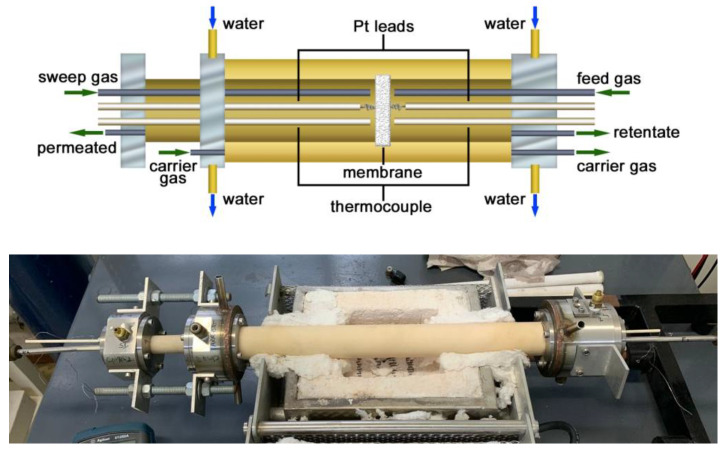
Top: schematics of the sample chamber for measuring gas permeation in a ceramic membrane; bottom: picture of the sample chamber positioned inside the split furnace.

Figure 2 shows details of input and output gases and water (for cooling the stainless-steel flanges) and a picture of the sample chamber positioned inside a split tubular furnace.

Two thermocouples are positioned close to each parallel face of the membrane to accurately monitor the temperature during permeation experiments. The feed gas is introduced into the sample chamber at the right side (see Figure 2, top) and is delivered close to the membrane surface through a thin alumina tube. Excess feed gas is exhausted through the outlet, labeled retentate. The sweep gas inlet is located on the opposite side, which also delivers gas close to the sample surface and carries the permeated gas to the mass spectrometer through the permeate outlet. The figure also shows the inlet and outlet of a third gas/gas mixture, identified as carrier gas, which can be used to support the possible removal of gas leakage.

The sample chamber is provided with Pt terminal leads for connecting both sides of the ceramic membrane to a 4192A Hewlett-Packard impedance analyzer (Yokogawa-Hewlett-Packard, Tokyo, Japan) connected to a series 360 Hewlett-Packard controller to collect electrical impedance spectroscopy data Z(ω) in the 10–10^7^ Hz frequency range. Z(ω) = [−Z″(ω) × Z′(ω)], where Z′ and Z″ are the real and the imaginary components of the electrical impedance; ω = 2 π f, f standing for the frequency of the input signal; Z(ω) is deconvoluted for the evaluation of the total electric resistivity with a special software [27]. Simultaneous electrochemical impedance spectroscopy and permeation data can be collected, making it possible to analyze, in situ, the membrane ionic conductivity, one of the parameters responsible for the efficiency of the membrane. Depending on the temperature of the measurement, the electrical resistivity of bulk and interfaces (grain boundary, second phases, and pores) of the ceramic membrane could be evaluated. The total electrical resistivity values, i.e., the sum of the electrical resistivity of bulk and interfaces, were used to obtain the Arrhenius plot, which is useful for determining the thermal activation energy of the solid electrolyte (membrane matrix) and the impregnated second phase (carbonates).

## 3. Results and Discussion

**Figure 3:**
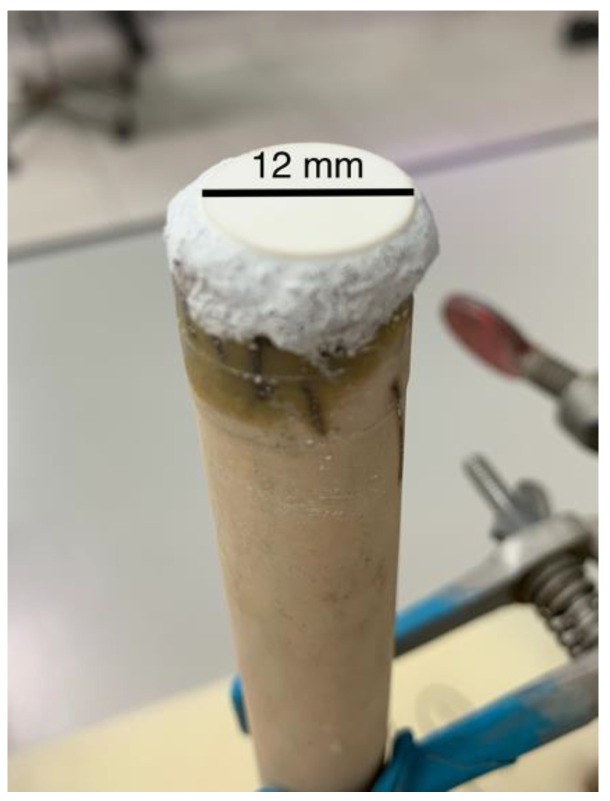
Picture of the dense alumina piece (white) cemented on top of the alumina tube of the sample chamber.

Before performing the permeation measurements, a leakage test was carried out using an alumina pellet instead of a ceramic membrane. Figure 3 shows a picture of a disk-shape dense alumina ceramic piece attached with cement to the alumina tube of the sample holder in the sample chamber.

**Figure 4:**
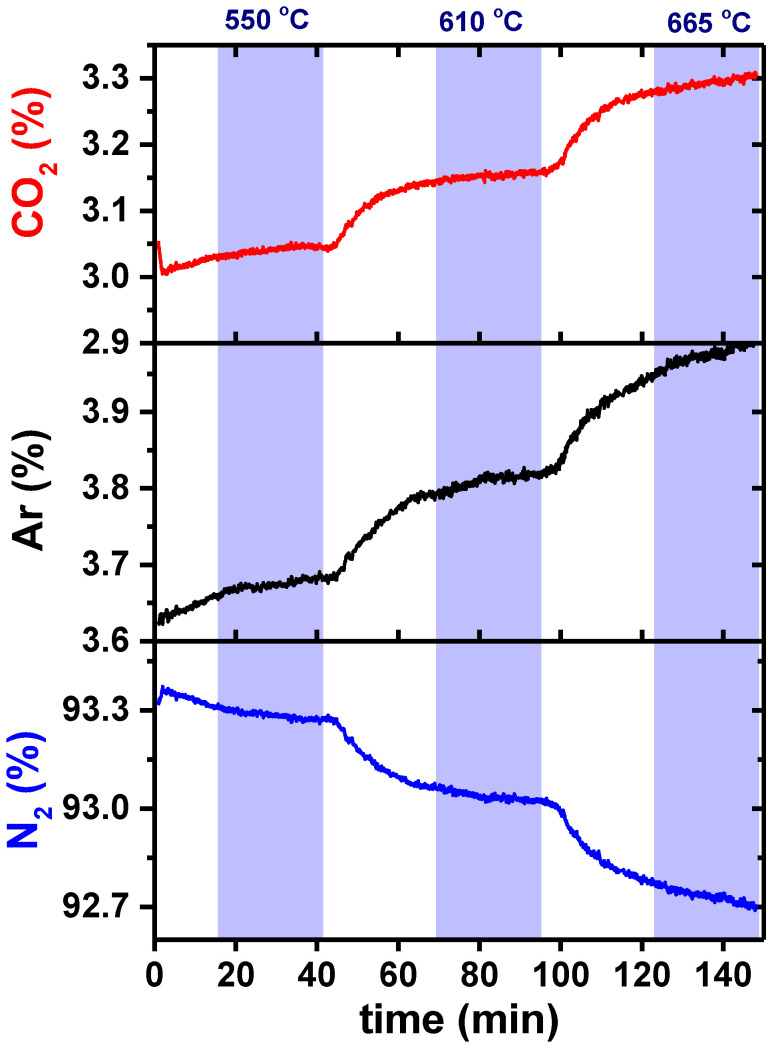
CO_2_, Ar, and N_2_ concentrations as a function of time at the permeated side of the setup shown in Figure 1. The colored areas represent the dwell time of the membrane temperature.

The dense alumina pellet was prepared to evaluate possible gas leakage through the ceramic sealant. For this test, an equal flux of carbon dioxide and argon was used as a feed gas, and nitrogen as a sweep gas, both at a 100 mL min^−1^ flow rate (*f_gas_*). The permeated gas (Cf. Figure 1) was analyzed with the mass spectrometer. The Ar, CO_2_, and N_2_ fluxes were evaluated at 550, 610, and 665 °C. These temperature values were selected for being close to the ones used during the permeation experiments (above 500 °C), so it was possible to analyze not only the amount of gas leaked, but also the proportion of the chemical species with increasing temperature. Figure 4 shows these results. Increasing the temperature leads to an increase in the mobility of CO_2_ (3.04 to 3.16 to 3.30%, respectively) and Ar (3.68 to 3.82 to 4.0%), increasing their content, as expected. The sweeping gas, N_2_, decreases from 93.3 to 93.0 to 92.7%, accordingly (Table 1).

Considering that the percentage of N_2_, detected by the mass spectrometer, represents 100 mL min^−1^ (same amount injected at the sweep side), Table 1 shows the measured average fluxes for CO_2_ and Ar collected at different temperatures.

An enhancement of Ar and CO_2_ contents is evaluated for increasing temperature. It is worth noting that, although the same amounts of CO_2_ and Ar were used as feed gas, the leakage of these two gases was not similar; these values should then be taken into account when analyzing the data collected after the permeation experiments. From Table 1 data, the evaluation of the ratio of CO_2_/Ar leakage to the permeated side yields close to 0.83 and does not change considerably with temperature.

After the gas leakage experiment, the three-atmosphere sample chamber was used to measure the CO_2_ permeation through a dual-phase ceramic-carbonate membrane (GDC-LNC). The temperature of the furnace was programmed to three temperature stages, the first one below the melting point of the carbonate mixture to evaluate sealing behavior of the ceramic glue, and the other two temperatures above 500 °C to measure the effective permeation; the feed gas was a mixture of 50% CO_2_ and 50% Ar, and N_2_ was the sweep gas, both at a 100 mL min^−1^ flow rate (*f_gas_*).

**Figure 5:**
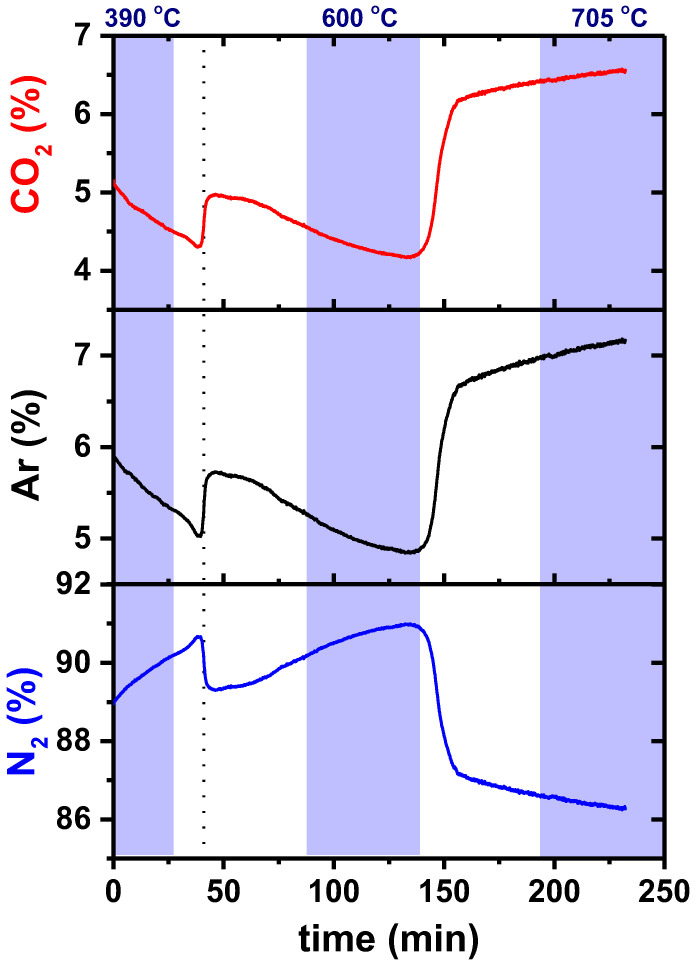
Percentage amounts of CO_2_, Ar, and N_2_ at the permeated side of the GDC-LNC ceramic membrane during a permeation experiment. The colored areas represent the period of membrane temperature stability and the vertical dotted line points to the time the membrane reaches 500 °C.

Figure 5 shows data of the CO_2_, Ar, and N_2_ concentrations measured with the mass spectrometer at the permeated side of the GDC-LNC membrane.

The measurement started at a temperature below the melting point of Li-Na carbonates, showing a significant increase in the contents of CO_2_ and Ar when the temperature of the membrane exceeds 500 °C. This effect is observable, assuming there is a chemical interaction of the ceramic sealant with the molten carbonates. Small portions of the carbonate end up being absorbed by the sealant, leading to self-healing of the gas leakage regions in the bulk of the sealant; this is favorable for the permeation process, but can also decrease the efficiency of the membrane, due to a decrease in the percentage of the carbonate phase in the bulk of the membrane.

There is a significant increment in the detection of CO_2_ and Ar for increasing the temperature to 700 °C. That increase shows that gas leakage also increased, since the ceramic membrane is not permeable to argon. There was probably an accumulation of molten carbonate at the membrane-ceramic adhesion paste interface, promoting open porosity.

Table 2 shows data of the contents of CO_2_ and Ar measured at three different temperatures. An evaluation of those values, which were detected with the mass spectrometer, did not allow for discriminating the CO_2_ permeated content from the leaked content; therefore, it was necessary to use the detected argon to determine the effective permeation.

Since the permeation occurs only at temperatures higher than the melting point of the Li-Na carbonate, the value obtained at 390 °C was used to determine the ratio (R) between CO_2_ and Ar leaked contents. This ratio is important to evaluate the fraction of CO_2_ detected with the mass spectrometer that was obtained through permeation, and the fraction resulting from the leakage. Since the ratio does not change considerably in this temperature range (as shown in the leakage test), the value detected at 390 °C was used to calculate the permeation above 500 °C.

The fraction (*P*) of CO_2_, Ar, and N_2_ detected at the permeated side and the total flow of the carrier gas (*f_gas_*) were used in the following equations to evaluate the permeation flux (*J*) of CO_2_ in mL cm^−2^ min^−1^ through the membrane [19]:

$$J_{CO_{2}total}=\frac{P_{CO_{2}}\cdot f_{gas}}{P_{N_{2}}\cdot \;S}$$

$$J_{CO_{2}leak}=\frac{R\cdot P_{Ar}\cdot f_{gas}}{P_{N_{2}}\cdot S}$$

$$J_{CO_{2}\;permeated}=J_{CO_{2}total}-J_{CO_{2}leak}$$

**Figure 6:**
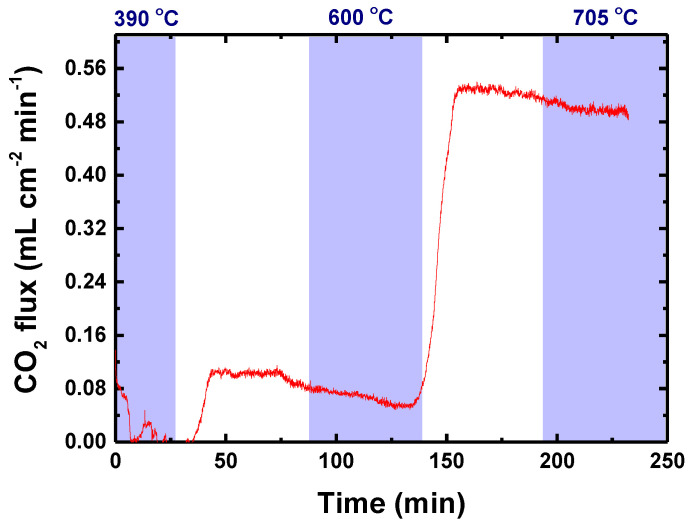
CO_2_ permeation flux of dense dual-phase GDC-LNC membrane with tape-casted porous support. The colored areas represent the period of membrane temperature stability.

*R* stands for the ratio between the CO_2_ and Ar concentrations detected below 500 °C at the permeated side, and S stands for the surface area of the membrane in contact with the feed gas. After using the above equations for the initial data obtained during the permeation experiments (Figure 5), the permeated CO_2_ could then be evaluated at 660 °C and 705 °C, Figure 6.

There is a negligible decrease in the permeation value during the isothermal stages, probably related to the interaction between the sealant and the molten carbonate, assumed above. Considering the active area of the membrane, the permeation flux of the membrane was estimated as 0.49 mL min^−1^ cm^−2^ and normalizing the partial pressure of CO_2_ on each side, the permeance of the membrane at 705 °C was estimated as 8.58 × 10^−8^ mol m^−2^ s^−1^ Pa^−1^.

Table 3 shows permeance data of some membranes with oxygen ion conduction under different experimental conditions, e.g., feed gas and operation temperature. The data obtained with our experimental setup are in the same range of results found in the literature, within 10^−8^–10^−7^ mol m^−2^ s^−1^ Pa^−1^.

**Figure 7:**
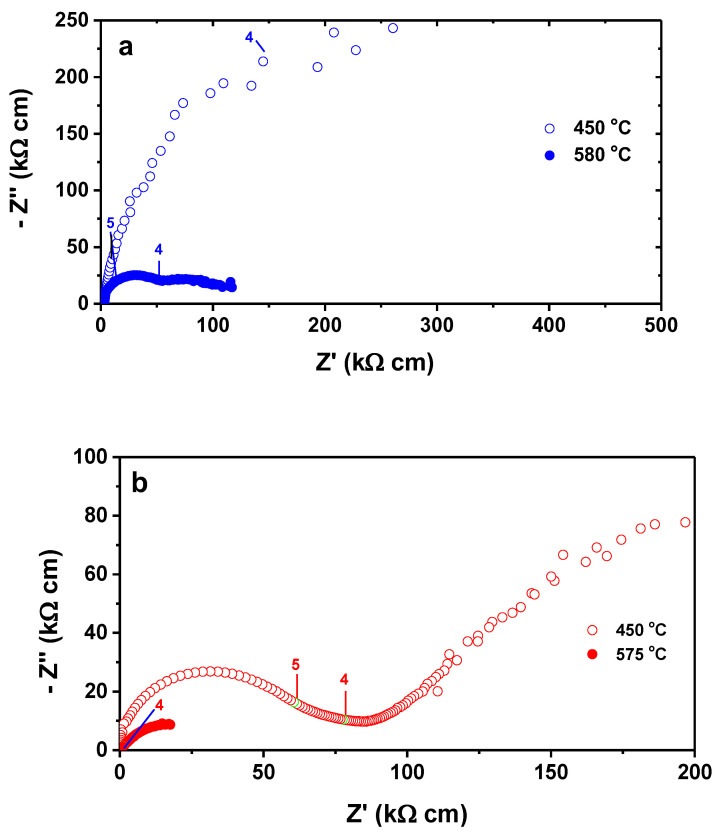
Impedance spectroscopy diagrams of CeO_2_: 20 mol.% Gd_2_O_3_ (**a**) porous and (**b**) infiltrated with 52 mol.% Li_2_CO_3_/48 mol.% Na_2_CO_3_, measured at temperatures below (450 °C) and above (575 °C) the melting point of 52 mol.% Li_2_CO_3_/48 mol.% Na_2_CO_3_. Numbers stand for log f (f:Hz).

Impedance spectroscopy measurements were performed on porous and infiltrated GDC membranes at temperatures below and above the melting point of the carbonate mixture to evaluate the electrical resistivity of the membrane over a wide temperature range. Figure 7 shows the impedance diagrams.

The electrical data, represented by impedance spectroscopy plots, show the resistive and capacitive behavior of porous CeO_2_: 20 mol.% Gd_2_O_3_ ceramic matrix (Figure 7a) and of the ceramic composite (porous matrix infiltrated with 52 mol.% Li_2_CO_3_/48 mol.% Na_2_CO_3_) (Figure 7b) at 450 °C and 580 °C, below and above the melting point of the eutectic mixture of lithium and sodium carbonates, respectively. The total electrical resistivity is higher in porous ceramics, as expected [32]. For the composite membrane, the contribution of the CO_3_^2−^ ions is predominant at 575 °C, Figure 7b.

**Figure 8:**
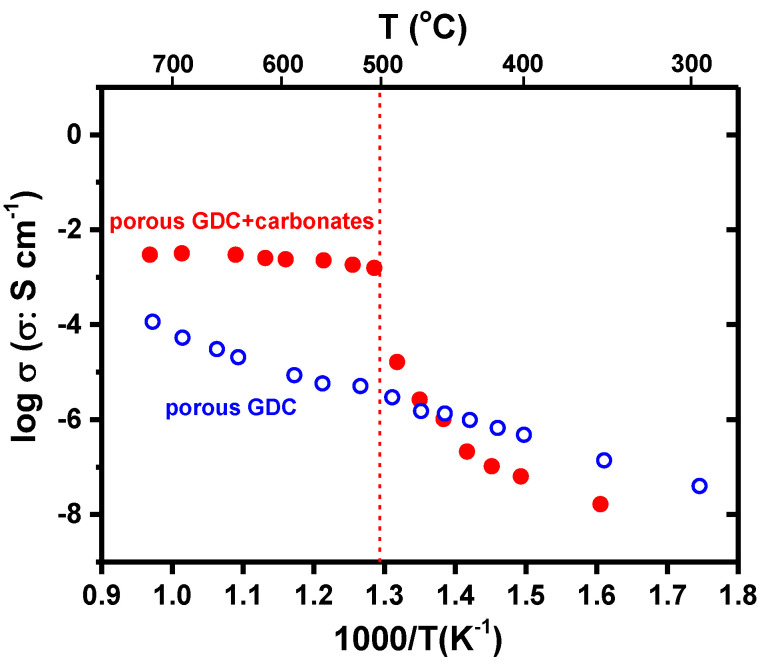
Arrhenius plots of the electrical conductivity of CeO_2_: 20 mol.% Gd_2_O_3_ porous (blue) and infiltrated (red) with 52 mol.% Li_2_CO_3_/48 mol.% Na_2_CO_3_.

Figure 8 shows Arrhenius plots of the total electric conductivity of porous gadolinia-doped ceria (GDC) before and after infiltration with the eutectic composition of lithium and sodium carbonates. The conductivity data of the porous membrane exhibits a linear behavior for the entire frequency range, as expected, with activation energy of 82.0 kJ mol^−1^, highlighting the conduction of oxide ions [33]. The sudden increase of the electrical conductivity of infiltrated membranes at 500 °C represents the contribution of the carbonate ion of the molten carbonate phase to the total conductivity of the composite ceramic pellet [13,27].

There is a significant increase in the total ionic conductivity of the infiltrated membrane (red dots) when the temperature approaches 500 °C; this was expected and occurs due to the fast increase of the ionic conductivity of the carbonate phase as a result of its melting. The thermal activation energy of the electrical conductivity for temperatures higher than the melting point of the carbonates was evaluated as 24 kJ mol^−1^. This value is typical for molten carbonates; the reported value for the activation energy of the conductivity of a eutectic mixture of lithium-sodium carbonates is ~19 kJ mol^−1^ [34]. The phase change measured near 500 °C sets the beginning of CO_2_ ionic transport through the membrane.

The total conductivity of the infiltrated membrane above 500 °C shows little variation since the conductivity of molten carbonate is nearly constant with increasing temperature. However, the total electrical conductivity of the ceramic phase (blue circles) keeps its linear increase during the entire temperature range. Although the increase in the electrical conductivity generated by the ceramic phase of the membrane is negligible, it is very important for the simultaneous ionic current of CO_3_^2−^ ions (through the molten carbonate phase) and O^2−^ ions (through the ceramic phase).

The knowledge of the electrical behavior of membranes contributes significantly to improving the design of the experimental setup, allowing for the collection of electrical data simultaneously with gas permeation.

## 4. Conclusions

An experimental setup was projected and assembled using an alumina sample chamber connected to mass flow controllers, a mass spectrometer, and an impedance analyzer. The sample chamber, capable of monitoring the temperature on both sides of the membrane and in situ electrochemical impedance spectroscopy measurements, was inserted in a split tubular furnace for high temperature testing of gas leakage and carbon dioxide ion permeation through a CeO_2_: 20 mol.% Gd_2_O_3_ porous ceramic infiltrated with a (Li,Na)_2_CO_3_ eutectic composition. The evaluated CO_2_ permeability data were in good agreement with data collected in the scientific literature. Impedance spectroscopy data were collected to evaluate the electrical conductivity of mobile ions at temperatures below and above the melting point of the alkali carbonates. The setup could also be used to evaluate the permeation of different gaseous species through other ceramic membranes with simultaneous monitoring electrochemical impedance spectroscopy behavior.

## Figures and Tables

**Table 1:** Gas fluxes measured with a mass spectrometer during the leakage test.

Temperature (°C)	Gas Flux (mL min^−1^)	
	CO_2_	Ar
550	3.2	3.9
610	3.4	4.1
665	3.6	4.3

**Table 2:** Gas fluxes measured with a mass spectrometer during the permeation test.

Temperature (°C)	Gas Flux (mL min^−1^)	
	CO_2_	Ar
390	5.1	6.0
600	4.6	5.4
705	7.5	8.3

**Table 3:** Permeance data obtained after testing several membranes (GDC: gadolinia-doped ceria; SDC: samaria-doped ceria; LSCF: lanthanum strontium cobalt ferrite; BYS: bismuth yttrium samarium oxide). Adapted from Ref. [19].

Ceramic Matrix	Feed Gas	Temperature (°C)	Permeance (mol m^−2^ s^−1^ Pa^−1^)	Ref.
GDC	20%CO_2_/15%H_2_O/62.4%Ar/2.6% H_2_	800 (heating)	5.35 × 10^−7^	[19]
		800 (cooling)	3.44 × 10^−7^	
SDC	5%H_2_/47.5%CO_2_/47.5%N_2_	700	8.56 × 10^−7^	[28]
LSCF	50%CO_2_/50%Ar	900	5.36 × 10^−8^	[15]
BYS	50%CO_2_/50%Ar	650	1.10 × 10^−8^	[29]
SDC	10%H_2_/45%CO_2_/45%N_2_	750	1.36 × 10^−7^	[30]
SDC	50%CO/35%CO_2_/10%H_2_/5%N_2_	900	1.68 × 10^−7^	[23]
SDC-BYS	49.5%CO/36%CO_2_/4.5%N_2_/10%H_2_	700	1.05 × 10^−7^	[31]
GDC	50%N_2_/50%CO_2_	650	4.40 × 10^−8^	[16]
GDC	50% Ar-50% CO_2_	705	8.58 × 10^−8^	This work

## Data Availability

Not applicable.
